# Using Gait Analysis to Evaluate Hip Replacement Outcomes—Its Current Use, and Proposed Future Importance: A Narrative Review

**DOI:** 10.3390/healthcare10102018

**Published:** 2022-10-12

**Authors:** Shayan Bahadori, Robert G. Middleton, Thomas W. Wainwright

**Affiliations:** 1 Orthopaedic Research Institute, Bournemouth University, Bournemouth, Dorset BH8 8EB, UK; 2 Orthopaedic Department, University Hospitals Dorset NHS Foundation Trust, Bournemouth, Dorset BH7 7DW, UK; 3 Physiotherapy Department, University Hospitals Dorset NHS Foundation Trust, Bournemouth, Dorset BH7 7DW, UK

**Keywords:** gait analysis, total hip replacement, functional recovery, new surgical technologies

## Abstract

Total hip replacement (THR) is one of the most common elective orthopaedic operations. However, evidence suggests that despite postoperative pain improvements, aspects of longer-term physical performance, such as walking ability, do not reach the levels expected when compared to the general population. Walking is best assessed by using gait analysis. This review aims to explain the concept of gait analysis, its use to evaluate THR outcomes, and its proposed future importance when evaluating new technologies proposed to improve functional recovery in individuals undergoing THR surgery. Furthermore, this review discusses the advantages and challenges of gait analysis in THR patients and provides recommendations for future work.

## 1. Introduction

Total hip replacement (THR) is one of the most common elective orthopaedic operations, and it is predicted to increase in prevalence as the population ages. The National Joint Registry (NJR) [1] reported that over the past three years, a total of 250,278 THR were undertaken in England, Wales, and Northern Ireland, and this figure is predicted to rise by 208% by the year 2035 [1]. Most THR are performed to treat end-stage osteoarthritis (OA), and hip OA commonly affects a patient’s function, causing difficulty in walking where altered gait biomechanics are observed. The literature has also revealed that despite postoperative pain improvements, the postoperative gait of these patients does not reach those of the general population, e.g., reductions in walking speed, stride length, sagittal hip joint range of motion (ROM), and peak hip abduction compared to the healthy population [2,3].

This review aims to explain the concepts of gait analysis and the current state of evidence in THR so that healthcare professionals not working in biomechanics on a day-to-day basis (i.e., clinical staff) can increase their knowledge. This will help them to understand the potential future importance of gait analysis when evaluating new technologies and approaches that attempt to improve functional recovery after surgery. Such approaches may include enhanced rehabilitation pathways and also new surgical technologies such as computer navigation, robotic-assisted surgery, patient-specific instruments (PSI), and new surgical approaches such as the direct anterior approach that propose a better functional recovery. 

Navigated THR surgery is an image-guided approach, usually incorporating a 3-dimensional preoperative planning simulator and intraoperative surgical navigator. Coupling optimised preoperative planning with accurate surgical navigation, this type of surgery assists the surgeon in properly orienting the component, minimising the risk of impingement and dislocations [4]. Robotic-assisted THR surgery is an evolution of navigated surgery where a robot helps the surgeon position or control the surgical tools to ensure that bone resection matches the planned operation [5]. First introduced in the 1990s, robotic-assisted THR provides accurate and reproducible component positioning and balancing of soft tissues [6]. In robotic-assisted THR, a preoperation computer tomography (CT) scan is used to preplan bony preparation to enhance the chance of optimal cup positioning and minimal deviation [6]. PSIs are an alternative to both navigation and robotic-assisted surgery for implant positioning. PSI systems are custom-made on a case-by-case basis, specific to the patient’s anatomy and the surgeon’s surgical plan. Using PSI, a 3D-printed model of patients’ anatomy will be printed and analysed by the surgeon to ensure the most appropriate implant and surgical approach [7,8]. 

Overall, all these new surgical technologies have a similar aim: to ensure a more accurate implant position. The implant insertion is suggested as a fundamental factor in the long-term outcome of a successful THR surgery [9]. It is evident that a poorly positioned cup correlates to increased surgical revision rates, joint instability, increased wear, and poor function [9]. A recent study also reported that after removing pain postsurgery, the main goal amongst patients undergoing THR surgery is to be able to walk freely [10]. Therefore, it is essential to prioritise the assessment of walking (or gait) as a functional activity after THR, so an understanding of the best possible technique to undertake this is required. 

## 2. Gait Analysis

Gait analysis assesses individuals with conditions affecting their ability to walk [11]. Walking is best assessed by evaluating the spatiotemporal gait parameters [11]. Spatiotemporal is one of three principle components of gait analysis, the other two being kinematic and kinetic [11]. The techniques used to perform gait analysis form a spectrum from simple and inexpensive at one end to complex and costly at the other. As a general rule, those which need little or no equipment are at the affordable side of the scale (i.e., visual observation and stopwatch) and are more commonly used in routine clinical settings.

In contrast, expensive laboratories with many pieces of equipment (gait labs with motion-capture cameras) are commonly used in university research settings or specialised clinical facilities. It is worth noting that the platform on which gait is assessed can be the most significant difference between these gait laboratories. Conventional gait analysis has historically utilised force plates and is performed overground, whereas more advanced gait systems now offer instrumented treadmills (with an integrated force plate) with the capability of self-selected walking speed. Instrumented treadmills are increasingly used in gait research and clinical environments, despite acknowledged limitations such as cost, requiring a designated facility, data variability, interpretation and time [12]. These treadmills offer potential advantages for advancing gait analysis in clinical and research settings by recording multiple consecutive strides in a small space [13]. Overall, three-dimensional optoelectronic movement measurement systems are the gold standard for analysing gait [14]. This technique consists of a series of retroreflective markers placed on the skin according to bony landmarks. Markers can be used to create a human body model and evaluate kinematic, kinetic, and spatiotemporal parameters movements. Spatiotemporal parameters are defined as distance-related parameters, which include step and stride length, and temporal parameters, defined as time-related parameters, which include stride time and walking speed [15]. Kinematic gait analysis studies the angular motion of the body, limbs, and joints during movement [15]. Kinetic gait analysis is the study of forces created by movement [15].

### 2.1. Gait Analysis in New Technologies for Total Hip Replacement Surgery

#### 2.1.1. Search Strategy

A computer-based search was completed in May 2022 using the mySearch Database (Bournemouth University). Articles published in the English language were reviewed. This included the Cochrane Database of Systematic Reviews library, CINAHL Complete^®^, Science Citation Index and Medline^®^. Search strategy terms were (gait analysis OR gait) AND (total hip replacement OR total hip arthroplasty OR hip replacement surgery OR THR) AND (robotic surgery OR navigation OR PSI). Only three articles [16,17,18] were found to evaluate the effect of new THR surgical technologies on gait.

#### 2.1.2. Results

All three studies reported a better precision in the placement of the implants with the robot’s assistance and presurgical planning. In two studies [16,17], outcomes of robotic-assisted THR were compared to conventional THR surgery and a healthy cohort. Despite using different protocols and techniques for the gait analysis, both studies reported no difference in the respective gait parameters six months after robotic surgery, in contrast to the gait of those who underwent THR surgery using conventional techniques. Bach et al. [16], also reported a significant reduction in kinematic hip extension in the robotic and conventional groups, as compared to the healthy group. Reininga et al. [17], findings suggested that although gait improved after surgery, small differences in several spatiotemporal parameters and angular movements of the trunk remained at 6 months postoperatively between both patient groups and healthy subjects. The third study was a case report by Watanabe et al. [18], describing a revision THR surgery of a 66-year-old woman using a computer tomography-based navigation system. This study aimed to use the navigation system to optimise the cup placement in the presence of a severe posterior pelvic tilt. This study reported that preoperative planning of implant orientation, and accurate placement of components, prevented dislocation in patients with severe posterior pelvic tilt. Overall, all studies reported a better precision in the placement of the implants with the robot’s assistance and presurgical planning. However, it is difficult to make a solid overall conclusion, as all three studies utilised different techniques for gait analysis and provided limited insight into the data processing methodology. Bach et al. [16] was the only study to outline the number of gait cycles collected. This study collected only five gait cycles and did not outline their justification for this number. Bach et al. [16] and Reininga et al. [17] both provided gait data at six months post-THR surgery, whereas the case report by Watanabe et al. [18] provided gait data at three years postsurgery time-point. An important limitation of all studies was their lack of kinetic gait data reports. 

In addition to these papers examining the effect of new THR technologies on gait, a recent review [19] concluded that gait patterns improve after THR compared to the preoperative state. However, there are deficits relative to healthy individuals. Regarding the differences relative to healthy subjects, walking speeds and spatiotemporal parameters were in many cases described as lower when compared with healthy individuals [19]. Furthermore, ROM is a crucial outcome in the recovery process after surgery. Yet, a significantly lower ROM is observed in comparison with the healthy subject even six months postconventional THR surgery [20,21,22]. It is suggested that this could be related to a load asymmetry between the operated and the healthy limb [19]. 

## 3. Discussion

This review aimed to explain gait analysis concepts and the current state of evidence in hip replacement to an audience with no or limited biomechanics knowledge and position its proposed future importance when evaluating new technologies to improve functional outcome measures. Gait analysis can provide necessary knowledge pre- and postop on the level of recovery of individuals post-THR surgery [23,24]. Whether with new technologies or conventional methods for THR surgery, gait assessment can also provide presurgical insight into surgeons on the best surgical approach and implant positioning [25,26].

Studies on surgical outcomes of robotic and navigation-assisted surgery provide relevant evidence to support the optimal cup positioning and higher accuracy in implant insertion in contrast to conventional THR surgical method [9,16,17]. However, evidence of its effect on gait improvement currently doesn’t exist. Only three papers were found through our systematic search, one of which was a case report of only one patient. It is worth noting that none of the studies reported kinetic gait parameters. Kinetic data can provide information on crucial abnormal gait, such as Trendelenburg gait [27], which can be observed in many patients pre-THR due to defective hip abductor muscles [28].

Furthermore, kinetic data could detect aberrant force transmission across a joint which may be associated with implant wear [28]. Variable methodology, lack of sufficient gait cycles [29], and different study designs reported in three studies made generalisability of the findings difficult. However, the results on improved spatiotemporal and hip kinematic data align with other THR studies previously reported [3,23]. 

## 4. Recommendation for Future Research

**Figure 1:**
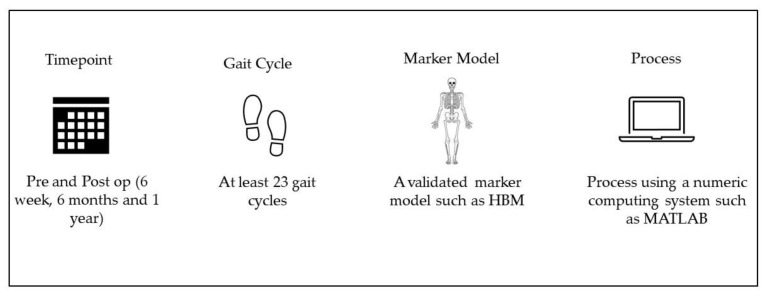
Our recommendations and clinical directions for gait analysis of THR patients.

As discussed, gait analysis can quantify movement patterns pre- and postoperatively. Still, a certain methodological quality should be considered when conducting a gait analysis. Several of our recommendations and clinical directions are as follows. First, we recommend that gait analysis is undertaken before and after surgery in order to understand individual gait changes. The first three months post-THR surgery is when a patient will experience accelerated improvement in their functional recovery, including gait [2]. Still, it is also essential to have a checkpoint beyond six months, as data have suggested that THR recovery declines after one year, and currently, there are not enough data on long-term gait for this cohort [3,30,31]. It is essential to acknowledge that some patients will find walking painful preoperation, and in many cases, they are afraid of falling [10]. This can now be better managed by new three-dimensional optoelectronic movement measurement systems such as the Gait Real-time Analysis Interactive Lab (GRAIL, Motekforce Link, Amsterdam, The Netherlands), with many safety features to avoid patient falling, such as handrails and harnesses. Secondly, it is recommended that 23 gait cycles should be captured to attain the characteristics of individual walk [29]. Thirdly, a validated marker placement model is essential. Van de Bogert et al. [32] have developed a complete human body model (HBM) that can be used to analyse three-dimensional kinematics, kinetics, and spatiotemporal gait assessment of orthopaedic-related patients. This model includes 25 reflective markers on the hip, knee, and ankle bony landmarks. Finally, the data process is an essential part of the gait analysis, and some systems offer an automatic process of gait assessment. Presurgery, most THR patients walk at a slow pace with a small step length. Subsequently, gait systems may not recognise an entire cycle due to a lack of full heel and toe contact. Therefore, it is recommended, especially for research centres, to export the raw data from their gait system and postprocesses using a numeric computing system such as MATLAB R2019a (The Mathworks Inc., Natick, MA, USA). Figure 1 illustrates our recommendation and clinical directions for gait analysis in THR populations.

## 5. Conclusions

This review concludes that gait analysis can be used to quantify movement patterns for both pre- and post-THR surgery and help better evaluate functional recovery in new surgical techniques such as robotic-assisted THR surgery. Currently, limited evidence suggests that despite better accuracy in implant positioning, there is no evidence of better spatial–temporal or kinematic gait data in a patient undergoing a robotic hip replacement in contrast to the conventional surgical method. Additionally, although gait was improved after surgery in each of these new surgical technologies, small differences in kinematic and spatiotemporal parameters were still reported at 6 months postoperatively between the THR patients and healthy individuals.

## Data Availability

Not Applicable.
